# Cost-effectiveness of inavolisib plus palbociclib/fulvestrant versus palbociclib/fulvestrant for PIK3CA-mutated advanced breast cancer in China

**DOI:** 10.3389/fphar.2026.1992102

**Published:** 2026-10-27

**Authors:** Zuojuan Xiang, Xiaojuan Chen, Ling Ma

**Affiliations:** 1 Department of Pharmacy, The Affiliated Cancer Hospital of Xiangya School of Medicine, Central South University, Hunan Cancer Hospital, Changsha, China; 2 Department of Clinical Pharmacy, The First People’s Hospital of Yunnan Province, The Affiliated Hospital of Kunming University of Science and Technology, Kunming, China

**Keywords:** breast cancer, cost-effectiveness, fulvestrant, inavolisib, palbociclib, PIK3CA

## Abstract

**Background:**

Based on the positive findings of the INAVO120 study, inavolisib is the only phosphatidylinositol 3-kinase alpha (PI3Kα) inhibitor approved for breast cancer treatment in China and has been included in the National Reimbursement Drug List following national medical insurance price negotiations.

**Methods:**

A partitioned survival model with a 30-year time horizon was constructed to assess the cost-effectiveness of inavolisib plus palbociclib/fulvestrant versus palbociclib/fulvestrant alone in patients with phosphatidylinositol-4,5-bisphosphate 3-kinase catalytic subunit alpha (PIK3CA)-mutated, hormone receptor (HR)+, human epidermal growth factor receptor 2 (HER2)-, locally advanced or metastatic breast cancer (a/m BC). Clinical data were extracted from the Asian cohort of the INAVO120 trial. Drug prices were sourced from the government procurement platform, while additional cost and utility parameters were derived from the published literature. The primary model outcome was the incremental cost-effectiveness ratio (ICER). To assess the robustness of the results, scenario and sensitivity analyses were performed. Additionally, a price simulation analysis was conducted for inavolisib.

**Results:**

The ICER was determined to be $36,932.91/quality-adjusted life-year (QALY), which exceeded the willingness-to-pay (WTP) threshold of $27,906/QALY. To attain cost-effectiveness, a further price reduction of 27.02% for inavolisib would be necessary. Scenario and sensitivity analyses confirmed the robustness of the results.

**Conclusion:**

The addition of inavolisib to palbociclib plus fulvestrant for PIK3CA-mutated HR+/HER2-a/m BC is not a cost-effective treatment strategy at the national level in China. However, this triple therapy may achieve cost-effectiveness in economically affluent regions with higher local WTP thresholds.

## 1 Introduction

Breast cancer is the most prevalent malignant tumor among women, leading in both incidence and mortality rates. In 2022, an estimated 2,295,686 new breast cancer cases and 665,684 cancer-related deaths were recorded globally (Bray et al., 2024). In China, 357,200 new cases and 75,000 deaths were reported (Han et al., 2024). Distinct disparities in the age distribution of breast cancer incidence exist between Eastern and Western countries. In the United States, the peak age for breast cancer incidence is 70 years, whereas in China and other East Asian countries, it is between 45 and 49 years (Yap et al., 2019). This pattern of early-onset incidence may be attributed to a birth cohort effect, which results from changes in risk factors across successive generations (Fan et al., 2014). This disparity highlights the significant disease burden faced by China.

Approximately 41.1% of patients with hormone receptor (HR)+/human epidermal growth factor receptor 2 (HER2)- advanced breast cancer harbor phosphatidylinositol-4,5-bisphosphate 3-kinase catalytic subunit alpha (PIK3CA) mutations (Anderson et al., 2020). These mutations are not only regarded as poor prognostic indicators but are also closely associated with resistance to endocrine therapy in patients with HR + breast cancer (Broege et al., 2025). Complex crosstalk and compensatory feedback mechanisms among the phosphatidylinositol 3-kinase-protein kinase B-mammalian target of rapamycin (PI3K-AKT-mTOR), estrogen-receptor, and cyclin-dependent kinase 4 and 6 (CDK4/6) signaling pathways indicate that targeting a single pathway may result in treatment resistance (Alves and Ditzel, 2023; Gao et al., 2024; Glaviano et al., 2024). Simultaneous inhibition of all three pathways may postpone resistance development and enhance the durability of therapeutic effects (Pascual et al., 2021; Tolaney et al., 2021). Inavolisib is a novel, potent, and highly selective inhibitor of PI3Kα, which also functions as a degrader of the PI3Kα mutant form. This dual mechanism of action confers superior antitumor efficacy and a favorable safety profile, facilitating its use in combination with other anticancer agents (Song et al., 2022). The phase III INAVO120 study (NCT04191499) was designed to assess the efficacy of inavolisib versus placebo in conjunction with palbociclib (a CDK4/6 inhibitor) and fulvestrant (a selective estrogen receptor degrader) in patients with PIK3CA-mutated HR+/HER2-locally advanced or metastatic breast cancer (a/m BC) (Jhaveri et al., 2025). The findings indicated that the triple therapy involving inavolisib demonstrated superior efficacy. Specifically, the median overall survival (OS) was 34.0 months in the inavolisib group versus 27.0 months in the placebo group (hazard ratio [HR] 0.67, 95% confidence interval [CI]: 0.48–0.94) and a median progression-free survival (PFS) of 17.2 months versus 7.3 months (HR 0.42; 95% CI: 0.32–0.55) (data-cutoff date: 15 November 2024). Meanwhile, it maintained a manageable safety profile. The findings suggested that inavolisib provided a superior treatment alternative for patients with PIK3CA mutations (Jhaveri et al., 2025). It has been granted a Class I recommendation in the Chinese Society of Clinical Oncology (CSCO) guidelines for patients with endocrine therapy-resistant PIK3CA-mutated HR+/HER2-a/m BC.

Based on the positive findings of the INAVO120 study (Jhaveri et al., 2025), inavolisib obtained marketing approval in China in March 2025 and was subsequently incorporated into the National Reimbursement Drug List (NRDL) following national medical insurance negotiations in January 2026, with a price reduction of approximately 61%. This development substantially mitigated the financial burden on patients and improved clinical accessibility. However, existing pharmacoeconomic evaluations of inavolisib in China are limited to the pre-reimbursement market environment (Huang et al., 2026; Zhu et al., 2026). Moreover, the survival data utilized were sourced from the overall population cohort of the global multicenter INAVO120 trial, without accounting for heterogeneity across different ethnic populations. The Asian cohort demonstrated a median OS of 32.7 months in the inavolisib group compared to 27.0 months in the placebo group (HR 0.74, 95% CI: 0.43–1.27), and a median PFS of 14.8 months versus 6.8 months (HR 0.38; 95% CI: 0.24–0.59) (data-cutoff date: 15 November 2024) (Yap et al., 2026). Considering these factors, the present study was grounded in the current healthcare reimbursement framework, particularly the updated drug pricing landscape. We used clinical data from the Asian cohort of the INAVO120 trial to generate evidence more consistent with domestic clinical practice in China. This study aimed to evaluate the cost-effectiveness of inavolisib combined with palbociclib/fulvestrant versus palbociclib/fulvestrant alone for the treatment of PIK3CA-mutated HR+/HER2-a/m BC from the perspective of the Chinese healthcare system. The findings are intended to provide economic evidence to support the optimization of healthcare reimbursement policies and clinical prescribing practices.

## 2 Methods

### 2.1 Model structure

A partitioned survival model with a 30-year time horizon was constructed using TreeAge Pro 2022 to assess the cost-effectiveness of inavolisib plus palbociclib/fulvestrant compared to palbociclib/fulvestrant for patients with PIK3CA-mutated HR+/HER2-a/m BC. At model termination, the majority of patients had entered the death state. The model assumed that patients started in a PFS state, with potential transitions to either a progressive disease (PD) state or a death state, as illustrated in Figure 1. Survival data points were extracted from the OS and PFS curves of the Asian cohort using WebPlotDigitizer. Subsequently, the survival curves were reconstructed and extrapolated in R version 4.3.2 using the survHE and flexsurv packages. To fit and extrapolate the survival curves beyond the clinical trial’s observation period, various standard parametric functions were employed, including exponential, Weibull, gamma, log-normal, log-logistic, Gompertz, and generalized gamma (Guyot et al., 2012). The selection of the optimal distributions was guided by the Akaike Information Criterion (AIC) and the Bayesian Information Criterion (BIC), supplemented by visual inspection. The results are detailed in Supplementary Table S1 and Supplementary Figures S1–S4. Key parameters of the optimal distributions can be found in Table 1. The model used a 28-day cycle length consistent with the clinical trial dosing schedule. The primary outputs comprised total costs, quality-adjusted life-years (QALYs), and the incremental cost-effectiveness ratio (ICER). To align with the 2025 version of China Guidelines for Pharmacoeconomic Evaluations (CGPE), the willingness-to-pay (WTP) threshold was defined as twice the Gross Domestic Product (GDP) *per capita* for the year 2025, amounting to $27,906/QALY. In addition, the study was conducted in strict adherence to the Consolidated Health Economic Evaluation Reporting Standards (CHEERS) reporting guideline (Husereau et al., 2022), as detailed in Supplementary Table S2.

**FIGURE 1:**
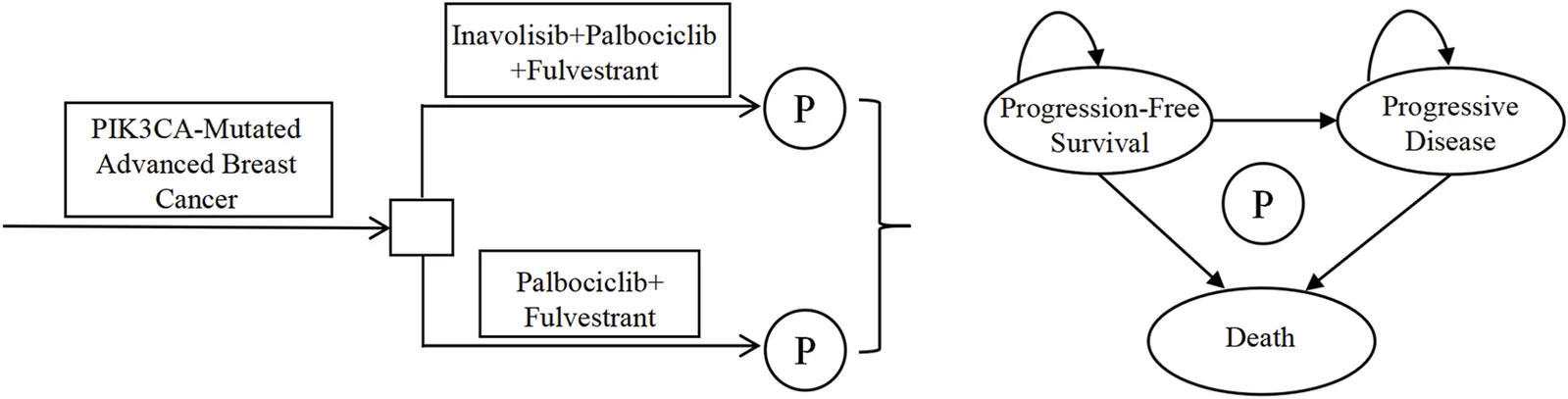
Diagram of the model structure. P, partitioned survival model.

**TABLE 1:** The key parameters of the model.

Variable	Baseline value	Minimum	Maximum	Distribution	Source
OS curve of inavolisib arm	shape = 1.820, scale = 35.382	​	​	Log-logistic	​
OS curve of placebo arm	meanlog = 3.392, sdlog = 1.198	​	​	Log-normal	​
PFS curve of inavolisib arm	meanlog = 2.820, sdlog = 1.138	​	​	Log-normal	​
PFS curve of placebo arm	shape = 1.622, scale = 6.879	​	​	Log-logistic	​
Cost ($)					
Tests per cycle	35.9	28.8	43.1	Gamma	(Lin et al., 2024)
Tumor assessment per unit	170.3	136.2	204.3	Gamma	(Liu et al., 2025)
Best supportive care per cycle	359.0	287.2	430.8	Gamma	(Liu et al., 2025)
Terminal care	1,494.0	1,195.2	1,792.8	Gamma	(You et al., 2024)
Inavolisib 9 mg/tablet	57.7	46.1	69.2	Gamma	(Hunan Healthcare Security Administration, 2026)
Palbociclib 100 mg	2.9	2.3	3.5	Gamma	(Hunan Healthcare Security Administration, 2026)
Fulvestrant 250 mg	37.8	30.2	45.4	Gamma	(Hunan Healthcare Security Administration, 2026)
Capecitabine 1,000 mg	0.6	0.5	0.7	Gamma	(Hunan Healthcare Security Administration, 2026)
Paclitaxel 100 mg	15.9	12.7	19.0	Gamma	(Hunan Healthcare Security Administration, 2026)
Neutropenia	84.5	67.6	101.4	Gamma	(You et al., 2024)
Hyperglycemia	155.0	124.0	186.0	Gamma	(You et al., 2024)
Stomatitis	6.3	5.0	7.5	Gamma	(Dranitsaris et al., 2015)
Thrombocytopenia	1,057.2	845.8	1,268.6	Gamma	(You et al., 2024)
Anemia	533.3	426.6	640.0	Gamma	(You et al., 2024)
Dose intensity (%)					
Inavolisib dose intensity in inavolisib group	95.4%	76.3%	100.0%	Beta	(Jhaveri et al., 2025)
Palbociclib dose intensity in inavolisib group	83.6%	66.9%	100.0%	Beta	(Jhaveri et al., 2025)
Fulvestrant dose intensity in inavolisib group	94.7%	75.8%	100.0%	Beta	(Jhaveri et al., 2025)
Palbociclib dose intensity in placebo group	84.7%	67.8%	100.0%	Beta	(Jhaveri et al., 2025)
Fulvestrant dose intensity in placebo group	94.1%	75.3%	100.0%	Beta	(Jhaveri et al., 2025)
Utility					
PFS	0.837	0.670	1.000	Beta	(Lloyd et al., 2006; Jia et al., 2025; Lang et al., 2025)
PD	0.443	0.354	0.532	Beta	(Lloyd et al., 2006; Jia et al., 2025; Lang et al., 2025)
Disutility due to grade ⩾3 AEs					
Neutropenia	0.150	0.120	0.180	Beta	(Wu et al., 2023)
Hyperglycemia	0.010	0.008	0.012	Beta	(Lloyd et al., 2006)
Stomatitis	0.150	0.120	0.180	Beta	(Liu et al., 2025)
Thrombocytopenia	0.110	0.088	0.132	Beta	(Yang et al., 2022)
Anemia	0.070	0.056	0.084	Beta	(Wu et al., 2023)
Risk of grade ⩾3 AEs (%)					
Neutropenia in inavolisib group	82.6%	66.1%	99.1%	Beta	(Jhaveri et al., 2025)
Hyperglycemia in inavolisib group	6.8%	5.4%	8.2%	Beta	(Jhaveri et al., 2025)
Stomatitis in inavolisib group	5.6%	4.5%	6.7%	Beta	(Jhaveri et al., 2025)
Thrombocytopenia in inavolisib group	13.7%	11.0%	16.4%	Beta	(Jhaveri et al., 2025)
Anemia in inavolisib group	6.8%	5.4%	8.2%	Beta	(Jhaveri et al., 2025)
Neutropenia in placebo group	80.4%	64.3%	96.5%	Beta	(Jhaveri et al., 2025)
Hyperglycemia in placebo group	0.0%	0.0%	0.0%	Beta	(Jhaveri et al., 2025)
Stomatitis in placebo group	0.0%	0.0%	0.0%	Beta	(Jhaveri et al., 2025)
Thrombocytopenia in placebo group	4.9%	3.9%	5.9%	Beta	(Jhaveri et al., 2025)
Anemia in placebo group	1.8%	1.4%	2.2%	Beta	(Jhaveri et al., 2025)
Body surface area, m^2^	1.62	1.30	1.94	Normal	(National Health Commission of the People’s Republic of China, 2021)
Discount rate (%)	4.5	0	5	Fix	CGPE

OS, overall survival; PFS, progression-free survival; PD, progressive disease; AE, adverse event; CGPE, china guidelines for pharmacoeconomic evaluations.

### 2.2 Patients

Patient characteristics were the same as those in the Asian subgroup of the INAVO120 trial, which focused on individuals with PIK3CA-mutated HR+/HER2-a/m BC (Yap et al., 2026). The median age of the participants was 50 years in the inavolisib group and 52 years in the placebo group. The proportion of patients with body mass index ≥25.0 was 22.4% in the inavolisib group and 32.2% in the placebo group. These patients experienced disease recurrence or progression during or within 12 months following adjuvant endocrine therapy. Each treatment cycle lasted 28 days. The inavolisib group was treated with inavolisib (9 mg orally daily, days 1–28), alongside palbociclib (125 mg orally daily, days 1–21) and fulvestrant (500 mg intramuscularly on day 1 and day 15 of cycle 1, then every 4 weeks). The placebo group received palbociclib and fulvestrant at identical dosages. Treatment in both groups continued until disease progression, intolerable toxicity, consent withdrawal, or other specified events occurred.

### 2.3 Cost and utility inputs

Only direct medical costs were considered, including drug costs, routine tests, tumor assessment, best supportive care, terminal care, and management costs associated with severe adverse events (AEs), as detailed in Table 1. Consistent with the INAVO120 trial protocol, tests per cycle included a 12-lead electrocardiogram, as well as assessments of hematology, blood chemistry, and urinalysis. Tumor assessments were conducted every 8 weeks during the first 2 years and every 12 weeks thereafter. The model included treatment-related costs associated with managing grade 3 or higher AEs that occurred in more than 5% of any group. All AEs were assumed to occur during the first cycle. Because subgroup-specific AE incidence data were unavailable for the Asian cohort, AE rates from the overall population were adopted. It was assumed that best supportive care was consistently provided throughout the treatment period, and that all patients received end-of-life care prior to death. The drug prices were obtained from the government procurement platform, among which the prices for palbociclib, fulvestrant, capecitabine, and paclitaxel were collected from the most recent centralized procurement bid results (Hunan Healthcare Security Administration, 2026). For a drug with multiple manufacturers, the average price was calculated based on a specified dose. The total drug cost was further estimated according to the actual consumption dosage in the model. The actual consumption dosage was calculated by multiplying the standard usage amount by the dose intensity. Drug wastage was not considered in this study. Additional costs were extracted from existing literature1 (Lin et al., 2024; Liu et al., 2025; You et al., 2024; Dranitsaris et al., 2015). The PFS and PD utility values in this study were elicited from the UK general public using the standard gamble method (Lloyd et al., 2006; Jia et al., 2025; Lang et al., 2025). Disutility caused by severe AEs was also incorporated into the model to comprehensively assess the impact of treatment safety on cost-effectiveness (Wu et al., 2023; Lloyd et al., 2006; Liu et al., 2025; Yang et al., 2022). Based on data from the INAVO120 study and recommendations from the CSCO guidelines, we assumed that patients received capecitabine or paclitaxel treatment after disease progression. In this model with a 28-day cycle length, paclitaxel treatment was limited to a maximum of 5 cycles, and capecitabine treatment was limited to a maximum of 8 cycles (Varnier et al., 2025). Detailed treatment regimens are provided in Supplementary Table S3. Costs were converted to U.S. dollars at an exchange rate of 1 USD = 7.1429 RMB, and all costs other than drug costs were adjusted to the 2025 level using the Consumer Price Index. According to the CGPE, both cost and utility values were subjected to an annual discount rate (DR_a_) of 4.5%. The discount rate for the 28-day cycle was ((1 + DR_a_)^(28/365)) - 1.

### 2.4 Sensitivity analysis

Sensitivity analyses, both one-way and probabilistic, were performed to assess the model’s robustness. For one-way sensitivity analysis, each key parameter was varied within a ±20% range of its baseline value (Zhu et al., 2026) or per the ranges reported in published references to evaluate how individual variables influenced model outcomes, as shown in Table 1. In contrast, the probabilistic sensitivity analysis entailed assigning appropriate probability distributions to each variable and performing 1,000 Monte Carlo simulations to examine the cumulative impact of concurrent variations in all variables on the model results.

### 2.5 Scenario analysis

In scenario 1, we adopted clinical data from the overall population in the INAVO120 trial. Patients were enrolled from 28 different countries, and the proportion of the Asian population was 38.2%. In scenario 2, it was assumed that patients did not use drugs acquired through centralized procurement; instead, they used originator drugs, which were considerably more expensive than their centrally procured counterparts, as detailed in Supplementary Table S4. Since there was a possibility that the PD and PFS utility values used in the study already accounted for AE-related impacts, we excluded AE-related disutility values in Scenario 3. In scenario 4, an alternative set of utility values sourced from another comparable study was employed. Specifically, a PFS utility value of 0.82 and a PD utility value of 0.55 were used (Liu et al., 2025). The PFS utility value was evaluated using the EQ-5D scale within the context of the Chinese population (Shen et al., 2022; Liu et al., 2025). In scenario 5, we employed suboptimal distributions to fit and extrapolate the survival curves, aiming to assess the impact of fitting and extrapolation uncertainties on model outcomes. The distributions and key parameters used in this scenario are presented in Supplementary Table S5.

### 2.6 Pricing analysis of inavolisib

In this analysis, ICER values were calculated as the price of inavolisib was systematically reduced. Furthermore, cost-effectiveness acceptability curves were constructed to determine the likelihood of the inavolisib triple therapy being considered cost-effective. To more comprehensively illustrate the influence of inavolisib pricing on economic evaluation outcomes, this analysis utilized not only the WTP threshold established in the base-case analysis but also adopted a more rigorous WTP threshold equal to the GDP *per capita* according to CGPE, amounting to $13,953/QALY.

## 3 Results

### 3.1 Base-case and scenario analysis

As indicated in Table 2, the base-case analysis revealed that the placebo group accrued costs amounting to $29,741.84, yielding 2.38 QALYs. In contrast, the inavolisib group incurred costs of $53,444.43 and achieved 3.02 QALYs. Consequently, the ICER was determined to be $36,932.91/QALY, which exceeded the WTP threshold, suggesting that inavolisib triple therapy was not an economically preferable strategy. In the scenario analysis, employing survival data from the overall population yielded an ICER of $35,315.68/QALY. Under the assumption that all patients used the originator drugs, the ICER was determined to be $45,629.23/QALY. Additionally, ICER values computed without incorporating the disutility values associated with AEs, or by employing alternative sets of utility values or suboptimal distributions, also surpassed the WTP threshold.

**TABLE 2:** Results of base-case and scenario analyses.

Treatment	Total cost ($)	Incremental costs ($)	QALYs	Incremental QALYs	ICER ($/QALY)
Base-case anlysis					
Placebo group	29,741.84	-	2.38	-	-
Inavolisib group	53,444.43	23,702.59	3.02	0.64	36,932.91
Scenario analysis 1					
Placebo group	29,177.13	-	2.42	-	-
Inavolisib group	53,274.58	24,097.45	3.10	0.68	35,315.68
Scenario analysis 2					
Placebo group	37,315.86	-	2.38	-	-
Inavolisib group	66,599.52	29,283.67	3.02	0.64	45,629.23
Scenario analysis 3					
Placebo group	29,741.84	-	2.51	-	-
Inavolisib group	53,444.43	23,702.59	3.18	0.67	35,457.35
Scenario analysis 4					
Placebo group	29,741.84	-	2.71	-	-
Inavolisib group	53,444.43	23,702.59	3.17	0.46	50,997.87
Scenario analysis 5					
Placebo group	29,011.90	-	2.32	-	-
Inavolisib group	49,946.29	20,934.39	2.71	0.40	52,935.32

QALYs, quality-adjusted life-years; ICER, incremental cost-effectiveness ratio.

### 3.2 Sensitivity analysis

Figure 2 displays the 15 variables with the greatest influence on the one-way sensitivity analysis outcomes. PFS utility, PD utility, and the cost of inavolisib notably influenced the model’s results. However, the ICER value consistently exceeded the WTP threshold. Figure 3 depicts the cost-effectiveness acceptability curve derived from the probabilistic sensitivity analysis, demonstrating that the probability of the inavolisib triple therapy being cost-effective increased as the WTP threshold was elevated. Furthermore, the scatter plot in Figure 4 shows that at a WTP threshold of $27,906/QALY, the probability of the inavolisib triple therapy being cost-effective was 11.80%.

**FIGURE 2:**
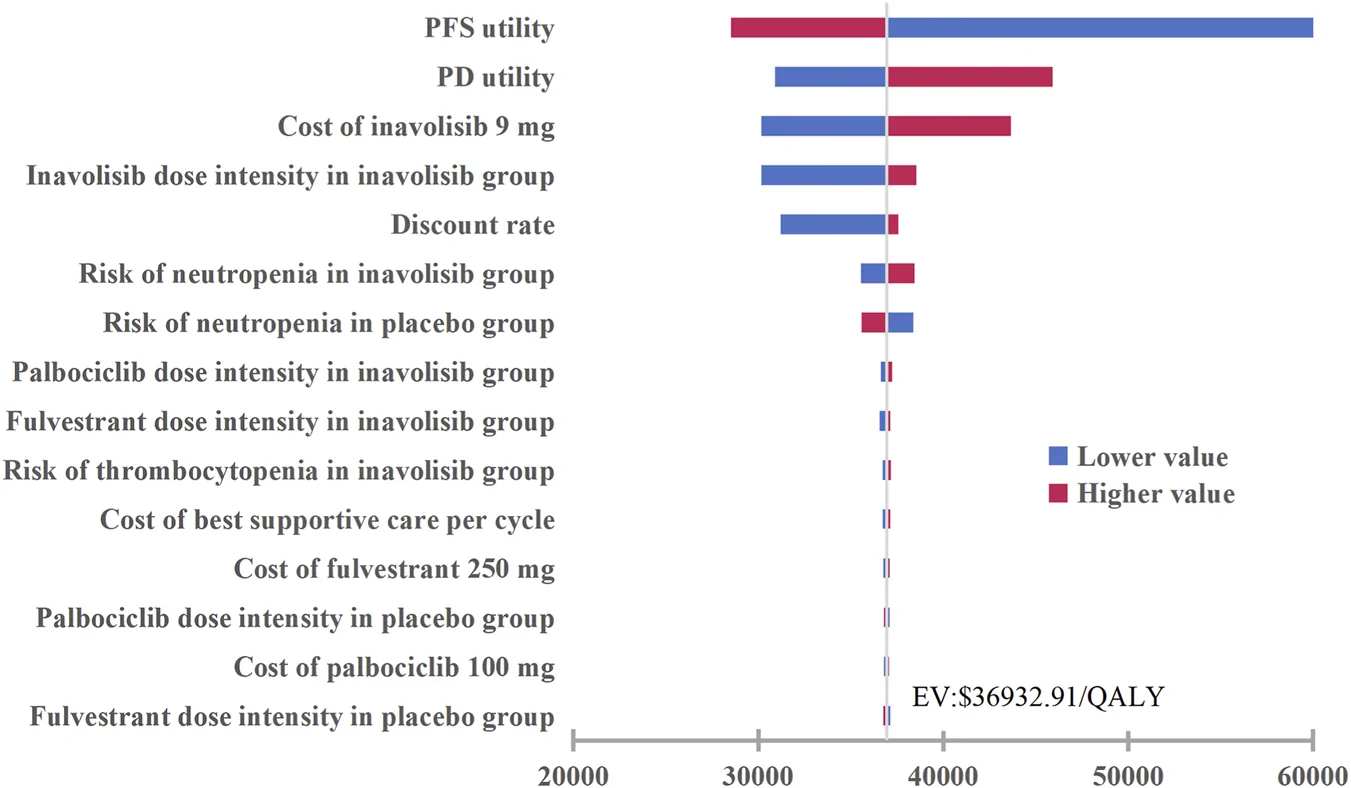
Tornado diagram of one-way sensitivity analysis. PFS, progression-free survival; PD, progressive disease; AE, adverse event.

**FIGURE 3:**
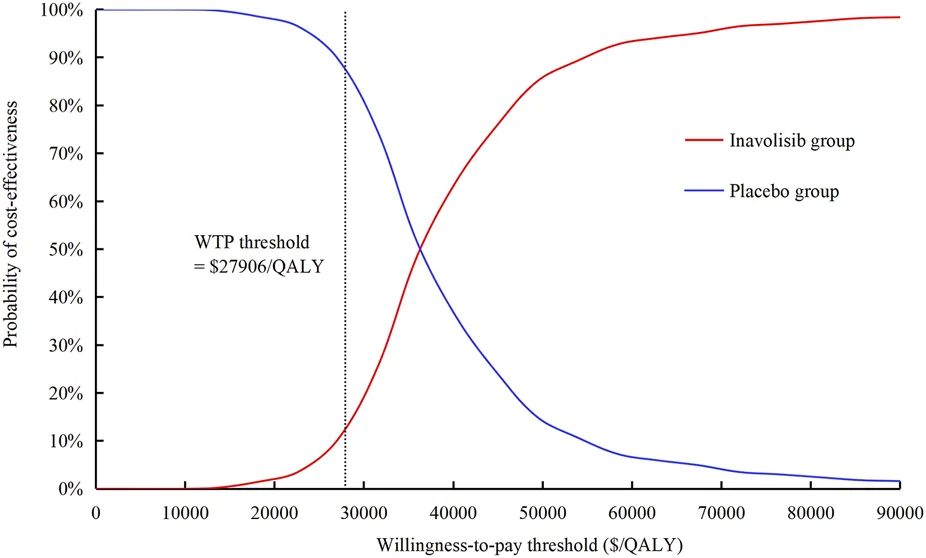
Cost-effectiveness acceptability curve in the base-case analysis. WTP, willingness-to-pay; QALY, quality-adjusted life-year.

**FIGURE 4:**
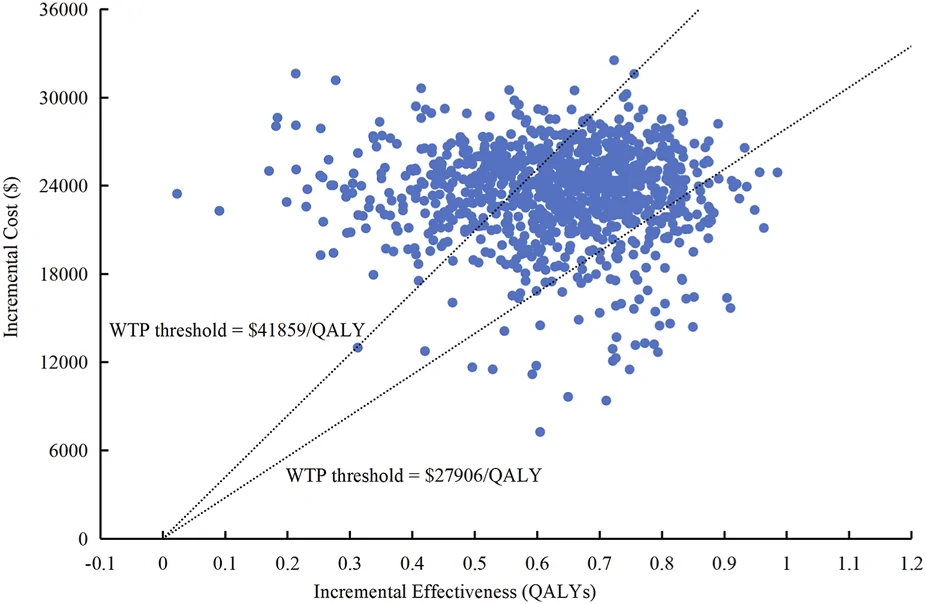
Scatter plot of the probabilistic sensitivity analysis. WTP, willingness-to-pay; QALY, quality-adjusted life-year.

### 3.3 Pricing analysis of inavolisib

As shown in Figure 5, the ICER value demonstrated a gradual decrease with the reduction in the price of inavolisib. Specifically, when the price reduction reached 27.02%, the ICER equaled the WTP threshold of two times the GDP *per capita*. If the WTP threshold was set equal to the GDP *per capita*, a price reduction exceeding 68.24% was required for the ICER to fall below this threshold. The cost-effectiveness acceptability curves corresponding to price reductions of 10%, 20%, 30%, and 40% for inavolisib are depicted in Figure 6.

**FIGURE 5:**
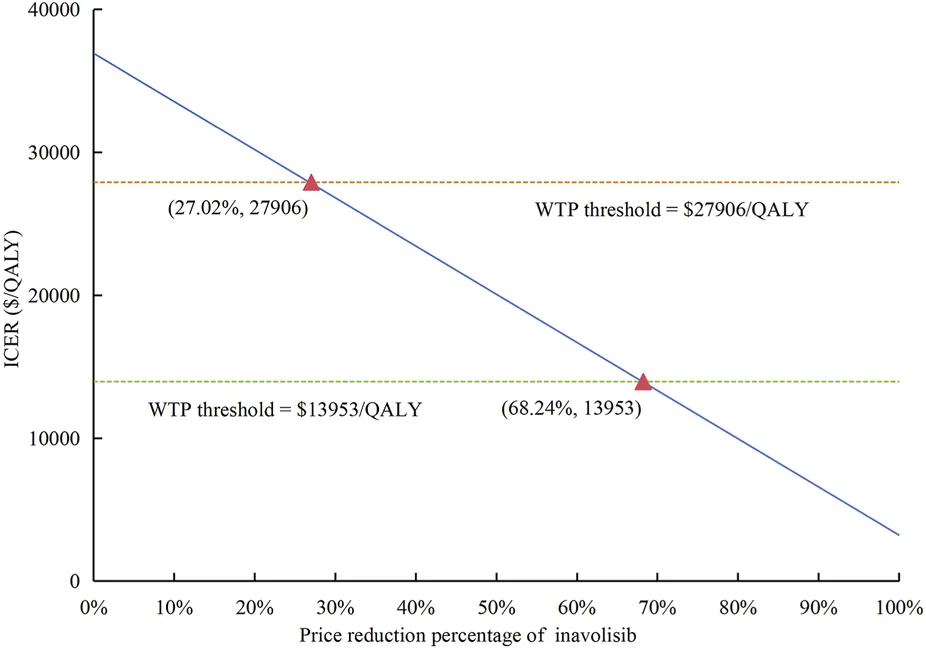
Results of the pricing analysis for inavolisib. QALYs, quality-adjusted life-years; ICER, incremental cost-effectiveness ratio.

**FIGURE 6:**
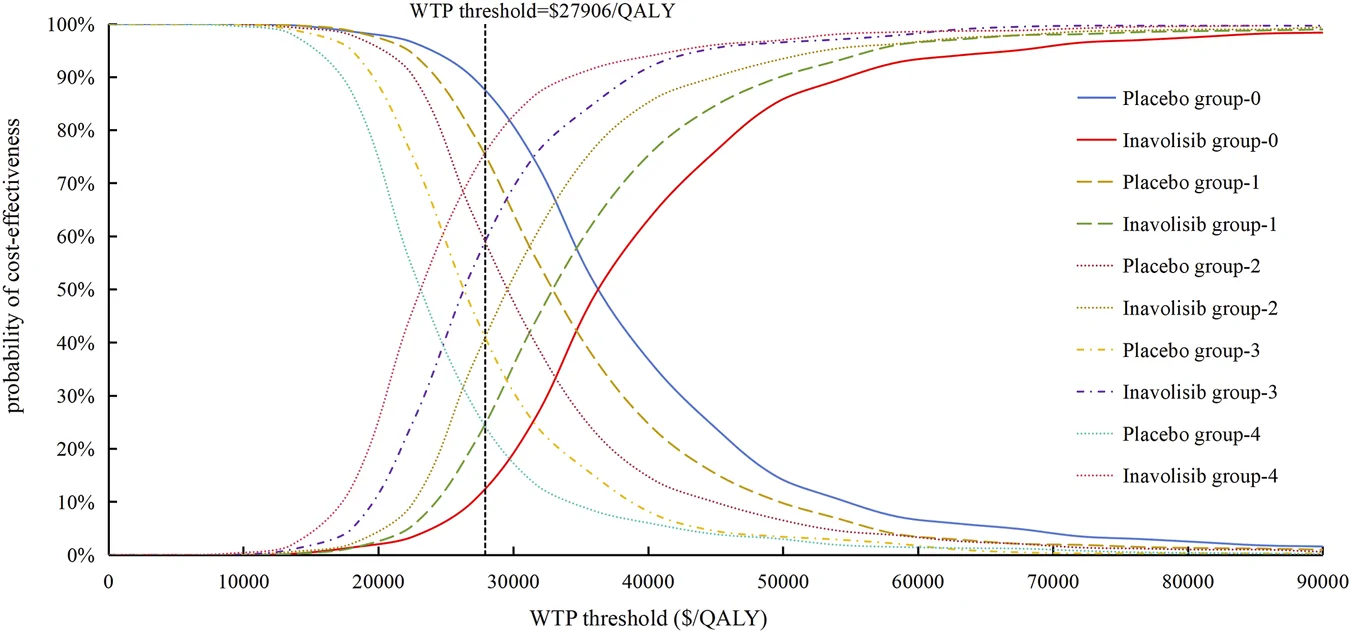
Cost-effectiveness acceptability curves conducted under a sequence of price reductions. 0–4 refer to the price reductions of 0%, 10%, 20%, 30%, and 40%. WTP, willingness-to-pay; QALY, quality-adjusted life-year.

## 4 Discussion

Inavolisib shows substantial efficacy and a well-characterized safety profile, which supports its combined use with palbociclib and fulvestrant to simultaneously inhibit three key pathways: the PI3K-AKT-mTOR, estrogen receptor, and CDK4/6 signaling pathways. This establishes inavolisib as superior to comparable agents such as alpelisib, making it the only approved PI3Kα inhibitor for breast cancer in China (André et al., 2019). Despite achieving notable improvements in survival benefit and being incorporated into the NRDL following national medical insurance negotiations, this study concluded that the combination of inavolisib with palbociclib/fulvestrant was still not cost-effective when compared to palbociclib/fulvestrant alone. Medical insurance reimbursement decisions encompass a multi-dimensional evaluation process that extends beyond mere cost-effectiveness analysis. Several other factors are integrated into the decision-making framework, including the magnitude of clinical benefit, unmet medical needs, the innovative value of the therapeutic agent, and findings from budget impact analysis (BIA). When the target patient population for the specified disease is relatively small and the associated incremental budgetary impact is manageable, it is possible to achieve a judicious balance between facilitating patient access to advanced therapies and maintaining the long-term fiscal sustainability of the medical insurance fund. Furthermore, according to the 2025 CGPE, for pharmaceuticals demonstrating exceptional value or substantial advancements in equity, a WTP threshold exceeding two times the GDP *per capita* may be adopted in practical research based on decision-making needs. Our model demonstrated that inavolisib triple therapy was cost-effective when the WTP threshold was elevated to three times the GDP *per capita*, equating to $41,859/QALY. The probabilistic sensitivity analysis indicated a 69.7% probability of inavolisib being cost-effective at this threshold, as illustrated in Figure 4. Additionally, considering regional economic disparities within China, the ICER in the base-case analysis already fell below the local WTP threshold of twice the GDP *per capita* in high-income regions, such as Beijing, Shanghai, Jiangsu, Fujian, and Zhejiang (National Bureau of Statistics of China, 2025), as shown in Table 3. Although precise regional cost-effectiveness assessments must consider variations in actual drug procurement prices and reimbursement policies across different provinces, this finding indicates that future health policy decisions should incorporate these regional affordability disparities, rather than relying exclusively on national average estimates.

**TABLE 3:** WTP thresholds across different provinces.

Province	WTP threshold	Province	WTP threshold
Beijing	63,886.38	Sichuan	21,653.11
Shanghai	60,798.84	Hainan	21,252.71
Jiangsu	44,994.05	Jiangxi	21,241.23
Fujian	38,617.37	Ningxia	21,066.23
Zhejiang	37,957.97	Xizang	21,135.39
Tianjin	36,999.82	Shanxi	20,655.20
Guangdong	31,120.69	Yunnan	18,931.25
Inner Mongolia	30,802.90	Qinghai	18,638.93
Hubei	28,792.79	Henan	18,168.53
Chongqing	28,252.67	Hebei	18,018.45
Shandong	27,320.84	Jilin	17,272.82
Shaanxi	25,176.05	Guizhou	16,431.70
Anhui	23,154.18	Guangxi	15,979.78
Hunan	22,742.86	Heilongjiang	15,148.47
Xinjiang	22,024.67	Gansu	14,790.91
Liaoning	21,905.95	​	​

WTP, willingness-to-pay.

The tornado diagram identified PFS utility, PD utility, and the cost of inavolisib as the most influential parameters. Consequently, we deduced that enhancing the quality of life associated with inavolisib triple therapy and implementing strategic pricing adjustments could be effective strategies for improving its cost-effectiveness profile. These findings are consistent with previous studies, in which the discount rate was also a prominent factor in the tornado diagram. This prominence was largely due to the fact that its plausible range was set at 0%–8% based on the 2020 version of the CGPE, whereas we employed a range of 0%–5% as recommended in the latest version (Huang et al., 2026; Zhu et al., 2026). These studies indicated that prior to its inclusion in the NRDL, the ICER of inavolisib triple therapy in China significantly exceeded the WTP threshold. In recent years, China has increasingly integrated innovative drugs into the NRDL through negotiation, thereby enhancing their accessibility, promoting equitable drug utilization among patients, and improving the overall standards of disease diagnosis and treatment. Our study demonstrates that even with its inclusion in the NRDL, the price of inavolisib would need to be reduced by a further 27.02% to keep its ICER values below the WTP threshold set at twice the *per capita* GDP. Nevertheless, the inclusion of inavolisib triple therapy for patients with endocrine therapy-resistant PIK3CA-mutated HR+/HER2-a/m BC within the scope of medical insurance reimbursement has significantly alleviated the treatment burden for these patients. However, considering the higher WTP threshold in the United States, inavolisib may be deemed cost-effective in that context (Liu et al., 2026). In addition, a study assessing the cost-effectiveness of alpelisib combined with fulvestrant versus fulvestrant alone in patients with PIK3CA-mutated, HR+/HER2-advanced breast cancer concluded that the alpelisib combination therapy was not cost-effective in the United States. The cost of alpelisib and PFS utility significantly impacted model outcomes (Wu et al., 2023). It should be noted that the 2025 version of the CGPE referenced in this study revises key parameters including the discount rate and WTP threshold to align with the current realities of China’s healthcare system. Furthermore, we incorporated clinical data from the Asian cohort to better reflect domestic clinical practice. We also accounted for the latest impacts of national medical insurance negotiations on inavolisib; all these elements represent key strengths of the present study.

Although the tornado diagram and scenario analyses demonstrated that utility-related variables were insufficient to alter the model’s outcomes, it remains essential to monitor the potential impact of specific AEs associated with inavolisib on quality of life. For instance, the overall incidence rates of hyperglycemia and stomatitis were significantly higher in the inavolisib group compared to the placebo group, at 63.4% versus 13.5% and 55.3% versus 28.8%, respectively, with grade 3 or higher AE occurrence rates at 6.8% versus 0% and 5.6% versus 0% (Jhaveri et al., 2025). Clinical experts suggested that for obese patients or those with prediabetes, it might be considered to initiate preventive metformin intervention 1–2 weeks prior to treatment. Furthermore, a dexamethasone-based, alcohol-free mouthwash was recommended for the prevention or treatment of stomatitis. Standardized AE management interventions are expected to enhance the quality of life for the inavolisib group (Im et al., 2026). Therefore, it is evident that integrating more comprehensive clinical trial data or real-world clinical practice data to refine model outcomes will be highly beneficial in the future.

The economic evidence associated with inavolisib in this study may offer valuable insights when considering further health decision-making and pricing strategy. However, this study also has several limitations. Firstly, the process of fitting and extrapolating survival curves could introduce uncertainty into the model. Moreover, employing a standard parametric model to fit survival curves with variable shapes and plateaued tails might lack the necessary flexibility to accurately capture inflection points (Shao et al., 2023). This highlights the critical need for future validation of the model using long-term follow-up data. Nonetheless, the application of suboptimal distributions in scenario analysis did not compromise the model’s outcomes, which partially attested to the model’s robustness. Secondly, owing to insufficient data on the Asian subgroup, AE incidence rates and dose intensity were derived from the overall population. This approach may introduce bias into the model. The model’s accuracy could be improved by incorporating Asian subgroup-specific clinical data to refine these parameters, subject to the availability of such data in the future. Thirdly, the Asian cohort of the INAVO120 trial has a limited sample size, and generalizing data from the Asian population to the Chinese population may also introduce bias. Fourthly, due to the lack of reliable utility values for advanced breast cancer in the Chinese population, the utility values for PFS and PD were derived from the UK general population. However, cultural perceptions of health and disease, as well as demographic and clinical characteristics, may vary significantly between Western and Chinese populations, potentially resulting in biased utility estimates. Although uncertainty analyses were performed on these utility values, it remains essential to validate the model using actual quality of life data in future research. Fifthly, considering that grade 1–2 AEs were easy to manage, the model only included costs and disutility values related to grade ≥3 AEs. However, one-way and scenario analyses confirmed that variables related to disutility values had minimal impact on the results.

## 5 Conclusion

Following inclusion in the NRDL, inavolisib plus palbociclib/fulvestrant remains not cost-effective as a therapeutic strategy for PIK3CA-mutated HR+/HER2-a/m BC at the national level in China. However, this regimen may be cost-effective in more affluent regions with higher WTP thresholds. Regionally differentiated medical insurance reimbursement policies and further targeted price optimization may help improve the cost-effectiveness and affordability of inavolisib.

## Data Availability

The original contributions presented in the study are included in the article/Supplementary Material, further inquiries can be directed to the corresponding author.
