# Supplementary material for: Cost-effectiveness of inavolisib plus palbociclib/fulvestrant versus palbociclib/fulvestrant for PIK3CA-mutated advanced breast cancer in China
Source: Front Pharmacol. 2026 Oct 27;17:1992102. doi: 10.3389/fphar.2026.1992102 (PMC13747866; doi:10.3389/fphar.2026.1992102)
Supplement: Supplementary file 1 [file DataSheet1.docx]

Supplementary Material

# 1 Supplementary Tables

**Supplementary Table 1.** Statistical goodness-of-fit for survival curves.

| **Survival curve** | **Exponential** | **Weibull** | **Gamma** | **Generalized gamma** | **Gompertz** | **Log-normal** | **Log-logistic** |
| --- | --- | --- | --- | --- | --- | --- | --- |
| OS curve of inavolisib arm |  |  |  |  |  |  |  |
| AIC | 235.059 | 232.321 | 232.030 | 234.004 | 234.504 | 233.316 | 231.599 |
| BIC | 237.119 | 236.442 | 236.151 | 240.185 | 238.625 | 237.437 | 235.720 |
| OS curve of placebo arm |  |  |  |  |  |  |  |
| AIC | 270.419 | 270.760 | 270.494 | 271.758 | 271.788 | 269.775 | 270.291 |
| BIC | 272.546 | 275.014 | 274.748 | 278.140 | 276.042 | 274.029 | 274.545 |
| PFS curve of inavolisib arm |  |  |  |  |  |  |  |
| AIC | 317.233 | 318.832 | 318.179 | 314.554 | 318.612 | 313.238 | 314.039 |
| BIC | 319.294 | 322.953 | 322.300 | 320.735 | 322.733 | 317.359 | 318.160 |
| PFS curve of placebo arm |  |  |  |  |  |  |  |
| AIC | 364.829 | 366.778 | 366.114 | 359.868 | 364.378 | 357.990 | 357.194 |
| BIC | 366.956 | 371.032 | 370.369 | 366.250 | 368.632 | 362.244 | 361.448 |

OS, overall survival; PFS, progression-free survival; AIC, Akaike Information Criterion; BIC, Bayesian Information Criterion.

**Supplementary Table 2.** CHEERS Checklist (2022)

| **Topic** | **No.** | **Item** | **Reported?** |
| --- | --- | --- | --- |
| **Title and abstract** |  |  |  |
| Title | 1 | Identify the study as an economic evaluation and specify the interventions being compared. | Yes |
| Abstract | 2 | Provide a structured summary that highlights context, key methods, results, and alternative analyses. | Yes |
| **Introduction** |  |  |  |
| Background and objectives | 3 | Give the context for the study, the study question, and its practical relevance for decision making in policy or practice. | Yes |
| **Methods** |  |  |  |
| Health economic analysis plan | 4 | Indicate whether a health economic analysis plan was developed and where available. | Not applicable |
| Study population | 5 | Describe characteristics of the study population (such as age range, demographics, socioeconomic, or clinical characteristics). | Yes |
| Setting and location | 6 | Provide relevant contextual information that may influence findings. | Yes |
| Comparators | 7 | Describe the interventions or strategies being compared and why chosen. | Yes |
| Perspective | 8 | State the perspective(s) adopted by the study and why chosen. | Yes |
| Time horizon | 9 | State the time horizon for the study and why appropriate. | Yes |
| Discount rate | 10 | Report the discount rate(s) and reason chosen. | Yes |
| Selection of outcomes | 11 | Describe what outcomes were used as the measure(s) of benefit(s) and harm(s). | Yes |
| Measurement of outcomes | 12 | Describe how outcomes used to capture benefit(s) and harm(s) were measured. | Yes |
| Valuation of outcomes | 13 | Describe the population and methods used to measure and value outcomes. | Yes |
| Measurement and valuation of resources and costs | 14 | Describe how costs were valued. | Yes |
| Currency, price date, and conversion | 15 | Report the dates of the estimated resource quantities and unit costs, plus the currency and year of conversion. | Yes |
| Rationale and description of model | 16 | If modelling is used, describe in detail and why used. Report if the model is publicly available and where it can be accessed. | Yes |
| Analytics and assumptions | 17 | Describe any methods for analysing or statistically transforming data, any extrapolation methods, and approaches for validating any model used. | Yes |
| Characterising heterogeneity | 18 | Describe any methods used for estimating how the results of the study vary for subgroups. | Not applicable |
| Characterising distributional effects | 19 | Describe how impacts are distributed across different individuals or adjustments made to reflect priority populations. | Not applicable |
| Characterising uncertainty | 20 | Describe methods to characterise any sources of uncertainty in the analysis. | Yes |
| Approach to engagement with patients and others affected by the study | 21 | Describe any approaches to engage patients or service recipients, the general public, communities, or stakeholders (such as clinicians or payers) in the design of the study. | Not applicable |
| **Results** |  |  |  |
| Study parameters | 22 | Report all analytic inputs (such as values, ranges, references) including uncertainty or distributional assumptions. | Yes |
| Summary of main results | 23 | Report the mean values for the main categories of costs and outcomes of interest and summarise them in the most appropriate overall measure. | Yes |
| Effect of uncertainty | 24 | Describe how uncertainty about analytic judgments, inputs, or projections affect findings. Report the effect of choice of discount rate and time horizon, if applicable. | Yes |
| Effect of engagement with patients and others affected by the study | 25 | Report on any difference patient/service recipient, general public, community, or stakeholder involvement made to the approach or findings of the study | Not applicable |
| **Discussion** |  |  |  |
| Study findings, limitations, generalisability, and current knowledge | 26 | Report key findings, limitations, ethical or equity considerations not captured, and how these could affect patients, policy, or practice. | Yes |
| Other relevant information |  |  |  |
| Source of funding | 27 | Describe how the study was funded and any role of the funder in the identification, design, conduct, and reporting of the analysis | Yes |
| Conflicts of interest | 28 | Report authors conflicts of interest according to journal or International Committee of Medical Journal Editors requirements. | Yes |

**Supplementary Table 3.** Treatment regimen following disease progression.

| **Drug** | **Usage and dosage** | **Rate** | |
| --- | --- | --- | --- |
|  |  | **Inavolisib group** | **Placebo group** |
| Capecitabine | 1000 mg/m² was administered orally twice daily from days 1 to 14, every three weeks. | 68.42% | 64.91% |
| Paclitaxel | 80 mg/m² was administered intravenously on day 1 of every week. | 31.58% | 35.09% |

**Supplementary Table 4.** The cost of originator drugs.

| **Originator drug** | **Manufacturer** | **Cost ($)** |
| --- | --- | --- |
| Palbociclib 100 mg | Pfizer Manufacturing Deutschland GmbH, Betriebsstatte Freiburg | 17.2 |
| Fulvestrant 250 mg | Vetter Pharma-Fertigung GmbH & Co. KG | 310.9 |
| Capecitabine 1000 mg | Roche Diagnostics GmbH | 5.8 |
| Paclitaxel 100 mg | Corden Pharma Latina S.P.A. | 231.5 |

**Supplementary Table 5.** The suboptimal distributions and key parameters employed in scenario 5.

| **Survival curve** | **Suboptimal** distribution | **Key parameters** |
| --- | --- | --- |
| OS curve of Inavolisib arm | Gamma | shape=1.806, rate=0.042 |
| OS curve of Placebo arm | Log-logistic | shape=1.477, scale=29.236 |
| PFS curve of Inavolisib arm | Log-logistic | shape=1.505, scale=16.461 |
| PFS curve of Placebo arm | Log-normal | meanlog=1.946, sdlog=1.078 |

OS, overall survival; PFS, progression-free survival.

# 2 Supplementary Figures

**Supplementary Figure 1.** Fitting and extrapolation of the OS curve in the placebo group. OS, overall surviva.

**Supplementary Figure 2.** Fitting and extrapolation of the OS curve in the inavolisib group. OS, overall surviva.

**Supplementary Figure 3.** Fitting and extrapolation of the PFS curve in the placebo group. PFS, progression-free survival.

**Supplementary Figure 4.** Fitting and extrapolation of the PFS curve in the inavolisib group. PFS, progression-free survival.
